# Multiscale Concurrent Topology Optimization and Mechanical Property Analysis of Sandwich Structures

**DOI:** 10.3390/ma17246086

**Published:** 2024-12-12

**Authors:** Zihao Li, Shiqiang Li, Zhihua Wang

**Affiliations:** 1 Institute of Applied Mechanics, College of Aeronautics and Astronautics, Taiyuan University of Technology, Taiyuan 030024, China; 2023310003@link.tyut.edu.cn (Z.L.); wangzh@tyut.edu.cn (Z.W.); 2 State Key Laboratory of Explosion Science and Safety Protection, Beijing Institute of Technology, Beijing 100081, China; 3 Shanxi Key Laboratory of Material Strength and Structural Impact, Taiyuan University of Technology, Taiyuan 030024, China

**Keywords:** multiscale concurrent topology optimization, micro-nano 3D printing, three-point bending test, numerical simulation, energy absorption

## Abstract

Based on the basic theoretical framework of the Bi-directional Evolutionary Structural Optimization method (BESO) and the Solid Isotropic Material with Penalization method (SIMP), this paper presents a multiscale topology optimization method for concurrently optimizing the sandwich structure at the macro level and the core layer at the micro level. The types of optimizations are divided into macro and micro concurrent topology optimization (MM), macro and micro gradient concurrent topology optimization (MMG), and macro and micro layered gradient concurrent topology optimization (MMLG). In order to compare the multiscale optimization method with the traditional macroscopic optimization method, the sandwich simply supported beam is illustrated as a numerical example to demonstrate the functionalities and superiorities of the proposed method. Moreover, several samples are printed through micro-nano 3D printing technology, and then the static three-point bending experiments and the numerical simulations are carried out. The mechanical properties of the optimized structures in terms of deformation modes, load-bearing capacity, and energy absorption characteristics are compared and analyzed in detail. Finally, the multiscale optimization methods are extended to the design of 2D sandwich cantilever beams and 3D sandwich fully clamped beams.

## 1. Introduction

With the rapid development of the mechanical engineering, automotive, and aerospace fields, practical engineering has put forward increasingly high requirements for the property of materials and structures. How to better achieve the integral design of material/structure and function [1,2] has been proposed by scholars in multiple fields, such as materials, mechanics, mathematics, and physics. Inspired by the natural biological structure, people have designed a large number of porous materials with high specific stiffness/strength, light weight, impact resistance, energy absorption, insulation, noise reduction, electromagnetic shielding, and other multifunctional characteristics by imitating the macro/micro multiscale porous configurations [3,4] of biological materials and structures [5,6,7]. However, these porous structures exhibit poor mechanical properties, and they tend to be ineffective when applied individually. These materials are often used as core layers in combination with metal or fiber panels to form sandwich structures with excellent structural properties and design ability. The panels provide strong bending and crushing resistance and mainly bear the effects of in-plane loads and bending moments. The core layer provides the normal stiffness and strength of the panel, withstands the shear stress generated by the compressive and transverse forces, and supports the panel to ensure its stability [8,9]. The emergence of this structure has greatly expanded the application range of lightweight porous materials. With the increasing maturity of additive manufacturing technology, it is possible to prepare various types of porous materials and structures with complex topological configurations, which have a broad range of applications in aerospace, high-speed trains, large transport equipment, and other military and civilian fields [10,11].

Topology optimization technology has been widely used in various industries [12,13]. Essentially, it is a numerical iterative process, which distributes materials in a fixed design reference domain to find the best material layout and optimize the objective function for a given set of boundary conditions. In order to design materials and structures that combine a variety of excellent properties, researchers in this field have developed numerous distinctive topology optimization algorithms, such as the Solid Isotropic Material with Penalization (SIMP) [14], the Evolutionary Structural Optimization (ESO) [15], Bi-directional Evolutionary Structural Optimization (BESO) [16,17], the Moving Morphable Component (MMC) [18], the Feature-drive Method [19], the Level Set Method (LSM) [20,21], the Independent Continuous Mapping Method (ICM) [22], the Feasible Domain Adjustment Method (FDM) [23], etc. Among them, the BESO method is widely used for optimizing materials and structures due to its simplicity and stable optimization process [24,25].

The micro topological configuration of porous materials is one of the key factors that affects the macro mechanical behavior of the materials and the structures. Compared with the parametric optimization and the shape optimization in a given design domain, the topology optimization has a larger design domain, which is a key and hot topic in the field of sandwich structure design [26]. With the gradual improvement of multiscale structural topology optimization design methods, it is possible to consider the heterogeneity of microscale materials in the pursuit of high-performance macrostructures. The ideal multiscale design should be a structure with optimal topology at both macroscales and microscales [27]. Therefore, the influence of the microscopic properties on the macroscopic properties must be considered in the optimization process. For sandwich structures, because the size of the characterizing element of the core layer is much smaller than the whole structure, the influence of the properties of the core layer structure on the macrostructure’s performance can be determined by using homogenization theory [28,29]. Therefore, the macrostructural/microstructural design variables can be concentrated into one system to achieve the multiscale optimal topological design of materials and structures [30].

However, the direct use of homogenization methods to calculate the effects of microstructures on the properties of macrostructures requires a large amount of computational resources. The topological configurations obtained based on homogenization methods will contain transition regions, which lead to unclear structures that are not conducive to the later fabrication and do not reflect the scale effects of the characterized elements. To solve these problems, they can be solved by improving algorithms based on homogenization methods. Zhang et al. [26] improved the computational efficiency using Kriging models based on homogenization methods. Sivapuram et al. [31] proposed a new multiscale optimization method to reduce the number of microstructures and optimize both materials and structures, which greatly saved the computational costs. Luo et al. [32,33] investigated the design and optimization of the microstructure of honeycomb composites based on the level set method. The optimization of multiple microstructures could largely improve the structural properties, but the interconnection between different microstructures is an essential issue. Wang et al. [34] focused on the interconnection between the multiple microstructures within the optimized structure. In order to obtain a clear configuration of the structure, Liu et al. [35] employed the penalty algorithms on the macroscales and the microscales, respectively, to carry out topology optimization design for the truss-like microstructure materials. The research found that the microscopic optimal topology configuration exhibited a truss-like structure. Wang et al. [36] investigated the design of gradient lattice structures with optimized fine-scale structures in additive manufacturing. The results showed the superior stiffness properties of the optimized graded lattice structure compared to the baseline design with uniform mesostructures. Liang et al. [26] applied multiscale optimization to the honeycomb sandwich structures, integrated the design of panel and core structures, and verified the effectiveness and excellence of the optimization results. Sigmund [37] used a projection method to obtain high-resolution, manufacturable structures from efficient and coarse-scale homogenization-based topology optimization results. The presented approach bridges coarse and fine scales such that the complex periodic microstructures can be represented by a smooth and continuous lattice on the fine mesh. Jiao Jia et al. [38] proposed a two-scale optimization model for heterogeneous structures with non-uniform porous cells at the microscale based on the HCA and extended it to 3D structural design. Guo et al. [39] used a 3D convolutional neural network to conduct 3D multiscale topology optimization, which saved computing costs and increased design flexibility.

The topological methods pursue the optimality of the results excessively, which usually makes the optimized structures precise and complex. Due to the existence of a microstructure, it is difficult to prepare the optimized results of the multiscale topology optimization structures using traditional processing methods. Additive manufacturing technology [11,40], through its integrated molding process, can perfectly present the results of topology optimization. The combination of topology optimization technology and additive manufacturing technology has overturned the limitations of traditional production technology, which opens up the design field and pushes the development of manufacturing. Many scholars have also proposed corresponding topology optimization algorithms to improve the accuracy of additive manufacturing [41]. Yu et al. present a hybrid topology optimization method for multipatch fused deposition modeling (FDM) 3D printing to address the process-induced material anisotropy [42]. Bi et al. studied the topology optimization of 3D concrete printing with various manufacturing constraints [43].

In summary, most of the literature on multiscale concurrent topology optimization algorithms is based on different optimization theories. The optimization processes and the ideas between different algorithms vary greatly, and the optimized results are not comparable, making it difficult to intuitively obtain the characteristics of various macro and microstructures. Meanwhile, due to the complexity of multiscale precision specimen manufacturing and the enormous workload of simulation calculations, further evaluation and comparison of the obtained optimized structures are usually not carried out. Therefore, based on the BESO method, combined with homogenization theory and periodic boundary conditions, this study proposes a simple and efficient optimization algorithm framework using the ABAQUS(6.14)-MATLAB(2019b) integrated optimization platform, which can achieve three different macro and micro optimizations (M, MM, MMLG). Different optimized specimens were prepared using high-precision 3D printing technology and compared through three-point bending tests. Simultaneously, finite element simulation was conducted using ABAQUS to analyze the deformation process of the macrostructure and the deformation mechanism of the microstructure, verifying the effectiveness and correctness of the optimization results. Finally, the algorithm was extended to the optimization of a sandwich cantilever beam and a 3D sandwich fully clamped beam.

## 2. Theoretical Analysis

### 2.1. Homogenization Theory

For the multiscale topology optimization, the effect of the microstructural property on the macrostructural property must be considered in the optimization process. For the sandwich structures, the microsize of the core layer is much smaller than the macrosize of the whole structure. The influence of the properties of the microstructure on the macrostructural performance can be determined by using the homogenization theory [29,44]. This theory can concentrate macrostructural design variables and microstructural design variables in one system [45] to realize multiscale topology optimization design of materials and structures. Two prerequisites are applied in this theory. 1. The macrosizes are much larger than that of the micro. 2. The microstructure exhibits a periodic distribution in the macrostructure.

**Figure 1:**
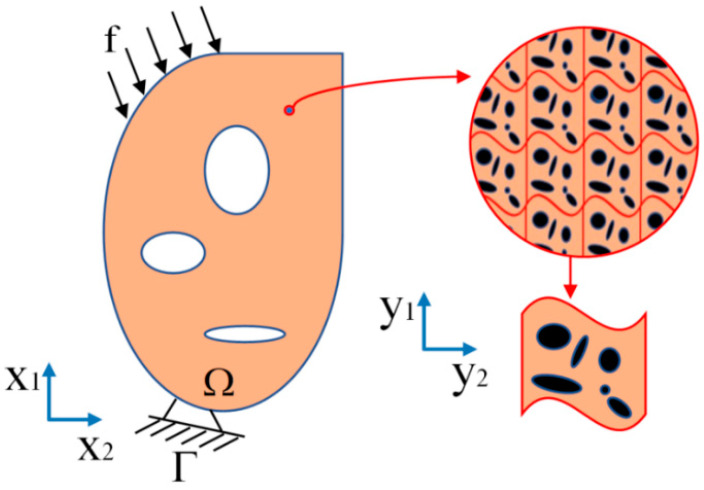
A 2D two-scale structure.

As shown in Figure 1, it is a schematic diagram of a 2D two-scale structure where Ω is the overall design domain of the structure, the external load acting on the structure is f, and the boundary conditions of the structure are Γ. Establish corresponding coordinate systems for the macroscopic and microscopic scales of the structure, where *x* is used to describe the macroscopic scale and *y* is used to describe the microscopic scale. The parameter ψ is the aspect ratio between the two scales, which is much less than 1. Firstly, the asymptotic expansion of the displacement field of the macrostructure can be expressed as [46]

(1) $$U^{\psi }(x)=U_{0}(x,y)+\psi U_{1}(x,y)+\psi^{2}U_{2}(x,y)+\cdot \cdot \cdot$$

The equivalent elastic modulus $D^{H}$ is served as a bridge between the macroscopic and the microscopic communication, which is determined by the porous form of the microstructure and reflects the main mechanical properties of the macrostructure. Considering the first-order variable of ψ only, without other discrete cases, it can be expressed as

(2) $$D_{ijkl}^{H}=\frac{1}{\left|\Omega_{m}\right|}\int_{\Omega_{m}}D_{ijpq}\left(\varepsilon_{pq}^{0(kl)}-\varepsilon_{pq}^{*(kl)}\right)d\Omega_{m}$$

where $\left|\Omega_{m}\right|$ is the microstructural area, $\varepsilon_{pq}^{0(kl)}$ is the linear independent unit test strain field ($\varepsilon_{11}^{0(kl)}=(1\;0\;0)$, $\varepsilon_{22}^{0(kl)}=(0\;1\;0)$, $\varepsilon_{12}^{0(kl)}=(0\;0\;1)$ for a 2D case), and $\varepsilon_{pq}^{*(kl)}$ indicates the unknown strain field in the microstructure, which can be solved using the following linear elastic equilibrium equation with y-period boundary conditions

(3) $$\int_{\Omega_{m}}D_{ijpq}\varepsilon_{pq}^{*(kl)}\frac{\partial v_{i}}{\partial y_{j}}d\Omega_{m}=\int_{\Omega_{m}}D_{ijpq}\varepsilon_{pq}^{0(kl)}\frac{\partial v_{i}}{\partial y_{j}}d\Omega_{m},\forall v_{i}\in H_{per}(\Omega_{m},R^{d})$$

where $v_{i}$ is the virtual displacement field in the displacement space $H_{per}$. To simplify the implementation of the topology optimization algorithm, based on the unitary mutual energy [37], the above equation is rewritten as

(4) $$D_{ijkl}^{H}=\frac{1}{\left|\Omega_{m}\right|}\int_{\Omega_{m}}D_{pqrs}\left(\varepsilon_{pq}^{0(ij)}-\varepsilon_{pq}^{*(ij)}\right)\left(\varepsilon_{rs}^{0(kl)}-\varepsilon_{rs}^{*(kl)}\right)d\Omega_{m}$$

In the FEA, the base cell is discretized into N finite elements, and the above equation can be approximated as

(5) $$D_{ijkl}^{H}=\frac{1}{\left|\Omega_{m}\right|}{\sum_{e=1}^{N}\left(u_{e}^{0\left(ij\right)}-u_{e}^{*\left(ij\right)}\right)}^{T}k_{e}\left(u_{e}^{0\left(kl\right)}-u_{e}^{*\left(kl\right)}\right)$$

where $\left(u_{e}^{0\left(kl\right)}-u_{e}^{*\left(kl\right)}\right)$ is the element displacement solution corresponding to the unit test strain field $\varepsilon^{0\left(kl\right)}$ and $k_{e}$ is the element stiffness matrix. So, the equivalent elastic modulus $D_{ijkl}^{H}$ can be expressed as

(6) $$D_{ijkl}^{H}=\left[\begin{array}{ccc} D_{1111} & D_{1122} & D_{1112}\\ D_{2211} & D_{2222} & D_{2212}\\ D_{1211} & D_{1222} & D_{1212} \end{array}\right]$$

### 2.2. Periodic Boundary Conditions (PBC)

**Figure 2:**
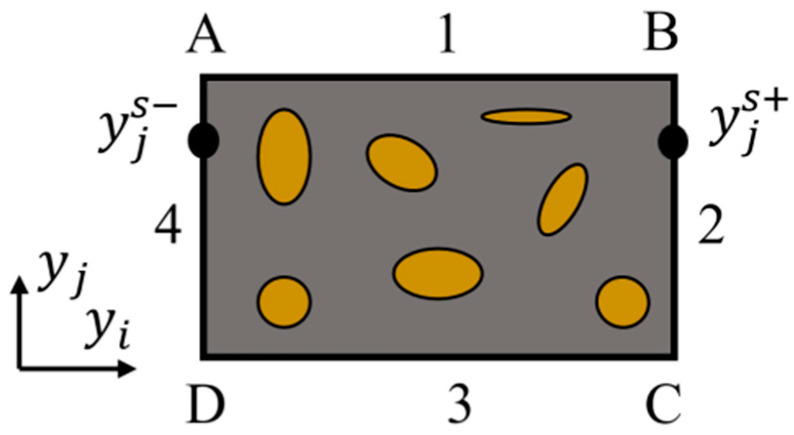
2D rectangular base cell model.

From the description of the homogenization method, it can be concluded that the macroscopic properties of the entire microstructure can be obtained by applying the method to the analysis of periodic base cells. While the deformation of the base cells in FEA is often complex, the periodic boundary conditions (PBCs) need to be introduced to satisfy the continuity and periodicity on the boundary of the microstructure. The corresponding nodes of the corresponding edges of the base cells are divided, as shown in Figure 2, with the superscript “$s^{+}$” and “$s^{-}$” denoting the opposite points on the two parallel boundaries, the + sign denoting the same direction as the coordinate axis, and the − sign denoting the opposite direction as the coordinate axis. Table 1 shows the results of the division, and the corresponding display displacement variables are constrained at the corresponding nodes. The boundary conditions of this form can act directly on the finite element model and satisfy the requirements of periodicity and continuity of displacements and stresses.

## 3. Multiscale Concurrent Topology Optimization Based on BESO

### 3.1. Multiscale Concurrent Topology Optimization Method

Taking the maximum macroscopic stiffness (minimum compliance) as the objective function, the optimal spatial distribution of the material microstructure is explored, and the optimization model is developed for a given target material volume. The mathematical model is described as follows:

$$Find:X_{i},X_{j}(i=1,2,\cdot \cdot \cdot m)(j=1,2,\cdot \cdot \cdot n)$$

$$Minimize:C=\frac{1}{2}F^{T}U$$

(7) $$\begin{array}{ll} Subject\;to: & V_{mac}=\sum_{i=1}^{m}V_{i}X_{i}\;X_{i}=\left\{X_{\min},1\right\}\\ & V_{\mathrm{mic}}=\sum_{j=1}^{n}V_{j}X_{j}\;X_{j}=\left\{X_{\min},1\right\} \end{array}$$

where F and U are the load and displacement of the macrostructure and C is the compliance of the macrostructure (strain energy). $V_{mac}$ is the target volume of the macrostructure, m is the total number of design domain elements, and $V_{i}$ and $X_{i}$ are the volume and relative density of the *i*th element in the macrostructure finite element model (design variables), respectively. $V_{mic}$ is the target volume of the microstructure, n is the total number of microscale elements, $V_{j}$ and $X_{j}$ are the volume and relative density of the *j*th element in the microstructure, respectively. In the BESO method, due to the “soft kill” method, $X_{\min}$ is used to represent the relative density of elements to be deleted with low sensitivity to prevent the singularity of stiffness matrix. The element still participates in the subsequent finite element analysis rather than being directly deleted, and it can be restored if the element has higher stress in the subsequent structural optimization. In this paper, $X_{\min}={1\;\times \;10}^{-9}\;g/cm^{3}$. It should be noted that the actual volume fraction $V^{r}$ of the material is jointly determined by the macroscopic and microscopic material properties

(8) $$V^{r}=V_{mac}\times V_{mic}$$

### 3.2. Sensitivity Analysis

By using the sensitivity filtering technology to weight the average of the sensitivity values of other elements within the filtering radius to correct the sensitivity of the central element, this can effectively reduce grid dependency and avoid differences in optimization results due to different grid sizes. The sensitivity analysis of multiscale topology optimization needs to be analyzed from both macroscales and microscales [47].

#### 3.2.1. Sensitivity Analysis at the Macroscale

Through the FEA of the macroscale elements, it can be concluded that

(9) F=KU

(10) $$K_{e}=\int_{V_{i}}B^{T}D^{H}BdV_{i}$$

where K is the entirety stiffness matrix, $K_{e}$ is the element stiffness matrix, B is the macroscopic structural displacement matrix, and $V_{i}$ is the volume of the *i*th element. The equivalent elastic modulus $D^{H}$ can be obtained from Equation (4). The local material inside of PBC is treated as isotropy, and its elastic modulus is assumed to be the interpolation function of element density

(11) $$D(X_{i})=D^{1}X_{i}^{p}$$

where *D*^1^ is the elastic modulus of the solid material. According to the adjoint variable method, it can be concluded that

(12) $$\frac{\partial D_{ijkl}^{H}}{\partial X_{i}}=\frac{p}{\left|\Omega_{m}\right|}\int_{\Omega_{m}}X_{i}^{p-1}D_{pqrs}^{1}\left(\varepsilon_{pq}^{0(ij)}-\varepsilon_{pq}^{*(ij)}\right)\left(\varepsilon_{rs}^{0(kl)}-\varepsilon_{rs}^{*(kl)}\right)d\Omega_{m}$$

At the macroscale, the sensitivity is calculated using compliance and design variables

(13) $$\alpha_{i}=\frac{\partial C}{\partial X_{i}}=-\frac{1}{2}U_{i}^{T}\frac{\partial K}{\partial X_{i}}U_{i}$$

where $U_{i}$ is the displacement vector of the *i*th element of the macrostructure.

#### 3.2.2. Sensitivity Analysis at the Microscale

Through the FEA of the microscale elements, it can be concluded that

(14) f=ku

(15) $$k_{e}=\int_{\Omega_{m}}b^{T}Dbd\Omega_{m}$$

where $k_{e}$ is the macroscale element stiffness matrix, *b* is the microscale element node displacement matrix, and *D* is the microstructure elasticity matrix. According to the SIMP method, the microstructure stiffness can also be expressed as an interpolation function of element density

(16) $$D\left(X_{j}\right)=D_{\min}+X_{j}^{P}\left(D_{0}-D_{\min}\right)$$

where $D_{0}$ is the elastic matrix of the material with the microstructural design variable $X_{j}=1$, $D_{\min}$ is the elastic matrix of the material with $X_{j}=X_{\min}$, and *P* is the penalty index. By using the adjoint variable method, the following can be obtained:

(17) $$\frac{\partial D^{H}}{\partial X_{j}}=\frac{1}{\left|\Omega_{m}\right|}PX_{j}^{P-1}\left(D_{0}-D_{\min}\right)u^{T}k_{e}u$$

Because $X_{j}$ is related to the response of all macroscale elements, the sensitivity of the microscale elements should be equivalent to the sum of the average compliance of all microscale elements [48]

(18) $$\alpha_{j}=\frac{\partial C}{\partial X_{j}}=-\frac{1}{2}\sum_{i=1}^{m}U_{i}^{T}\frac{\partial K_{e}}{\partial X_{j}}U_{i}=-\frac{1}{2}\sum_{i=1}^{m}U_{i}^{T}\int_{V_{i}}B^{T}\frac{\partial D^{H}}{\partial X_{j}}BdV_{i}U_{i}$$

### 3.3. Multiscale Concurrent Topology Optimization Process Based on BESO Method

**Figure 3:**
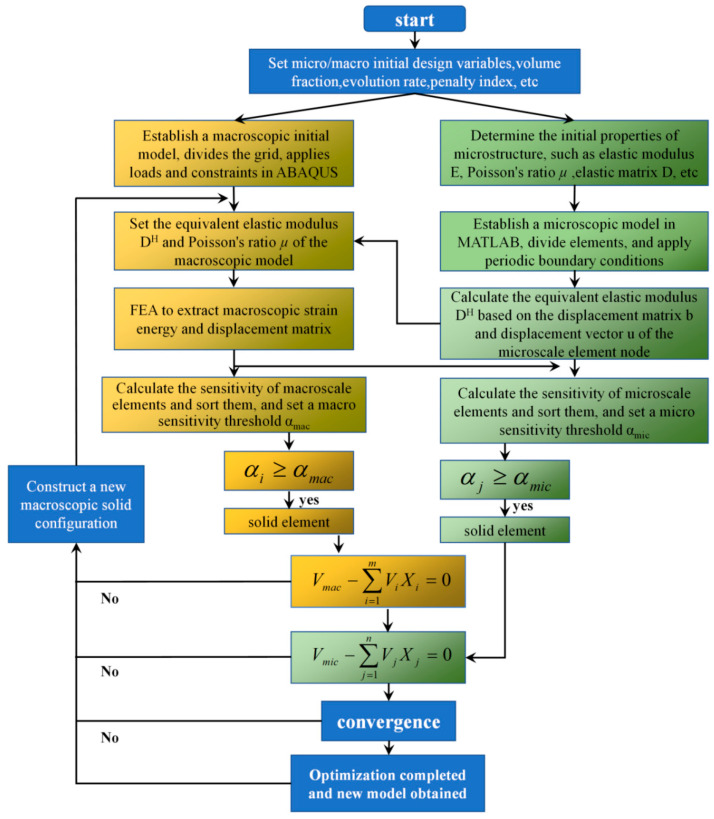
Flowchart of multiscale concurrent topology optimization.

The multiscale concurrent topology optimization process is shown in Figure 3.

1. Set initial design variables and optimization parameters, and provide macrostructural and microstructural target volume fraction, evolution rate, penalty index, and other parameters.

2. Establish a finite element macroscopic initial model using ABAQUS, discretize the design domain using finite element mesh, and apply loading and boundary conditions.

3. Determine the initial properties of the microstructure: elastic modulus *E*, Poisson’s ratio μ, elastic matrix *D*, etc. And, establish a microscopic model in MATLAB, divide the elements, and apply periodic boundary conditions.

4. Calculate the equivalent elastic modulus $D^{H}$ based on the node displacement matrix *b* and the displacement vector *u* of the microscale element.

5. Based on the calculation results of the 4th step, set the equivalent elastic modulus $D^{H}$ and Poisson’s ratio μ of the macrostructure.

6. The finite element analysis is carried out through ABAQUS, and the strain energy and displacement matrix of the macrostructure are extracted.

7. Calculate the sensitivity of macroscale and microscale elements separately and set sensitivity thresholds $\alpha_{mac}$ and $\alpha_{mic}$. If $\alpha_{i}\geq \alpha_{mac}$ or $\alpha_{j}\geq \alpha_{mic}$, the element is recognized as a solid element and enters step 8. If not, it is considered an “empty element” and will no longer participate in subsequent operations in this iteration.

8. Based on the calculation results of the 7th step, calculate the current entirety of the macroscopic and microscopic structure volumes and determine whether the set volume fraction and convergence conditions are met. The convergence conditions are shown in Equation (19). Loop iteration steps 3 to 8 and update variables until convergence conditions are met and optimization is completed. If not, construct a new macroscopic solid configuration and return to step 3.

(19) $$\frac{\left|\sum_{i=1}^{N}\left(C_{m-i+1}-C_{m-N-i+1}\right)\right|}{\sum_{i=1}^{N}C_{m-i+1}}\leq \varepsilon$$

where ε is the relative error value, which is taken as 0.01, and m is the number of iterations. Take a positive integer N to represent the average of N iterations, which avoids the problem of difficult convergence.

## 4. Multiscale Concurrent Topology Optimization of Sandwich Simply Supported Beams

**Figure 4:**
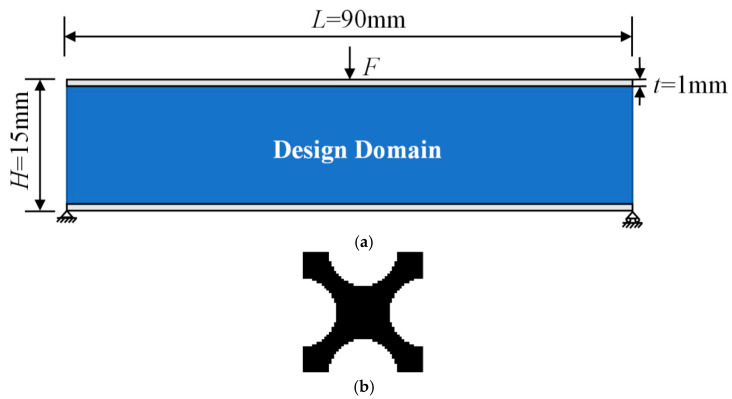
Optimization model and initial microstructure, (**a**) initial optimization model of sandwich simply supported beam, (**b**) initial microstructure.

As shown in Figure 4a, the optimized model is a 2D sandwich simply supported beam with length *L* = 90 mm and height *H* = 15 mm, which is subjected to a concentrated load at the center of the top surface of the structure. The top and bottom panels are the non-design domains, and the panel thickness *t* = 1 mm. The design domain is discretized into quadrilateral elements with a side length of 1 mm. During the optimization process, both the core layer and the top and bottom panels are set as ductile UV curable resin materials required for 3D printing for the later experimental preparation. To compare the various mechanical properties of the five structures, their masses (volume fractions) are controlled to be equivalent, while the three multiscale structures have the same initial microstructure. The initial microscopic configuration (Figure 4b) generally requires some pores to avoid the uniformly distributed sensitive field caused by the initial imposed periodic boundary conditions [49]. In this paper, an “X” shape with a volume fraction of 0.5 is used as the initial configuration. Due to the asymmetry of the macroscopic loads and the boundary conditions, the optimized microstructures are characterized by orthogonal anisotropy, which can better improve the macroscopic structural performance [50].

### 4.1. Macro and Micro Concurrent Topology Optimization

**Figure 5:**
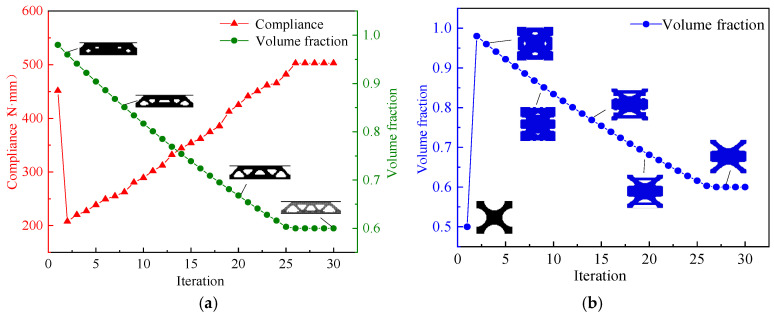
MM structure optimization history. (**a**) Macrostructure optimization history chart, (**b**) microstructure optimization history chart.

In the process of macro and micro concurrent topology optimization, the macrostructure is a truss-like configuration consisting of a series of microscale elements with the same topological structure arranged periodically. A microscale element is discretized into 50×50 quadrilateral elements, with a side length of 0.02 mm. The parameters set in optimization are as follows: macrostructural volume fraction *Macrovolfrac* = 0.6, microstructural volume fraction *Microvolfrac* = 0.6, penalty index *P* = 3, filtering radius *r_min_* = 3, and evolution rate *er* = 0.02. Figure 5a,b show the optimization processes of the macrostructure and the microstructure.

From Figure 5a,b, it can be seen that both macro and micro optimization of the MM structure converged after 30 iterations. Influenced by the initial configuration of the microstructure in Figure 5b, the compliance (strain energy) from step 1 to step 2 of the macro optimization process in Figure 4a changes sharply. The volume fraction of the initial microstructure “X” is 0.5, and it changes to 0.98 in the step 2. The solid part significantly increases, and the compliance decreases. The macrostructure and the microstructure are continuously optimized towards the target volume fraction of 0.6 in subsequent iterative steps. As the volume fraction gradually decreases, the compliance increases until the optimization is completed at the end of the iteration. The final value of compliance is 503.27 N⋅mm. Table 2 shows the results of macro and micro concurrent topology optimization.

### 4.2. Macro and Micro Gradient Concurrent Topology Optimization

This optimization is combined with the SIMP method and the BESO method. Firstly, the SIMP method is used to optimize the macrostructure with a known boundary condition to obtain the macroscopic material distribution. During the optimization process, due to the characteristics of the SIMP method, the intermediate density elements will be generated. Based on the traditional SIMP interpolation method (Equation (9)), the ordered interpolation of the elastic modulus of various materials is constructed [51].

(20) $$D(X_{i})=D^{1}F(X_{i})$$

where $F(X_{i})$ is an extended power function, which can be expressed as

(21) $$F(X_{i})=AX_{i}^{P}+B$$

**Figure 6:**
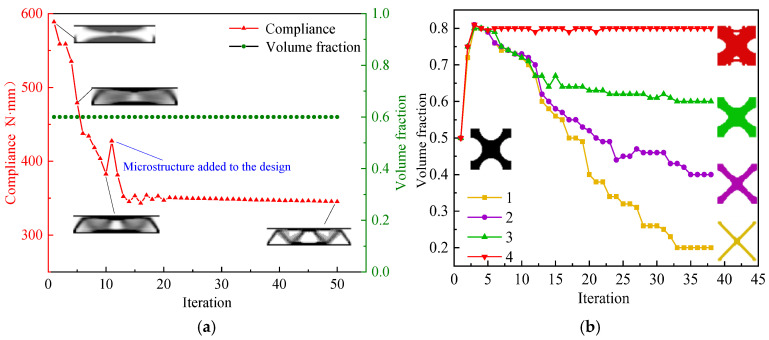
MMG structure optimization history. (**a**) Macrostructure optimization history chart. (**b**) Microstructure optimization history chart.

The scaling coefficient *A* and the translation coefficient *B* are given as

(22) $$A=\frac{D_{i}-D_{i+1}}{X_{i}^{P}-X_{i+1}^{P}}\;;\;B=D_{i}-AX_{i}^{P}$$

where $D_{i}$ is the elastic modulus of ordered material *i*, which is controlled using the ordered interpolation method to generate the ordered intermediate density elements to obtain different microscale element structures. Thus, the relative density of macroscale elements is considered as a volume constraint for microstructure design. In this way, concurrent topology optimization can be implemented in the macrostructure and the microstructure, while the position distribution of the microstructure in the macrostructure can be designed. Four ordered microstructures are used in this example, with volume fractions of 0.2, 0.4, 0.6, and 0.8. A microstructure is discretized into 50×50 quadrilateral elements. The parameters set in optimization are as follows: *Macrovolfrac* = 0.6, *Microvolfrac* =0.6, *P* = 3, *r_min_* = 3, *er* = 0.02. Figure 6a,b show the optimization processes of the macrostructure and the microstructure.

**Figure 7:**
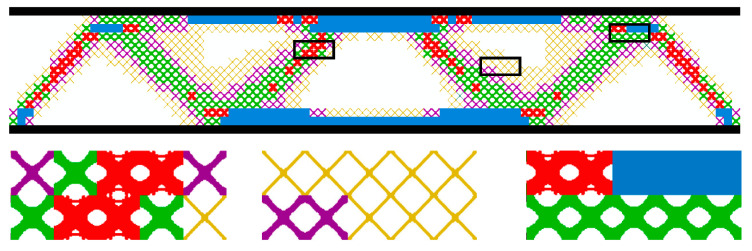
MMG structure topology optimization result.

From Figure 6a, the volume fraction of the macrostructure is always maintained at 0.6. The SIMP method is used in the first 10 iterations to optimize the macrostructure only, and the compliance decreases during the iterations. The subsequent 40 iterations are macrostructure and microstructure optimization concurrently. From step 11, four “X”-shaped microstructures with a volume fraction of 0.5 are added to the design (Figure 6b), which causes an increase in macroscopic compliance from step 10 to step 11. Subsequently, the volume fractions of the four microstructures increase sharply in the iterative step of the microstructure, and the compliance of the macrostructure decreases rapidly. Then, the volume fractions of the four representative microstructures start to gradually reach the preset values with the optimization process. During this process, the compliance tends to smoothly decrease after a small oscillation. Convergence is completed in step 50, and the final compliance is 342.15 N⋅mm. From the optimization results (Figure 7 and Table 3), it shows that microstructures with larger volume fractions are distributed along the main path of force transfer or the top and bottom edges of the structure. This structure can transmit a load and resist deformation more effectively.

### 4.3. Macro and Micro Layered Gradient Concurrent Topology Optimization

**Figure 8:**
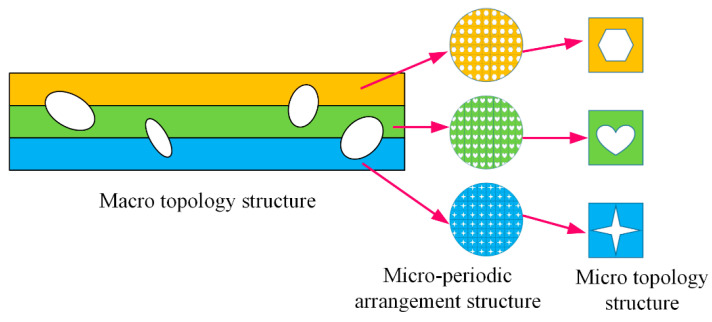
MMLG structure topology optimization.

As shown in Figure 8, this optimization is combined with the BESO method, which provides a new idea of concurrent topology optimization for multiscale structure design. The macrostructure is divided into a specified number of layers in a specified direction, and each layer consists of a periodic arrangement of the same topology microstructure. The microstructure between the layers has different configurations and volume fractions. A separate microstructural design is separated from the macrostructure layout by using independent microstructural design variables. To reduce the computational cost and the complexity of microscopic design constraints, the structural coverage constraint (SCC) and the average porosity constraint (APC) [52] are introduced:

(23) $$G_{SCC}=\sum_{l=1}^{L}\sum_{k=1}^{m}X_{k,l}V_{k,l}-f_{1}\left|\Omega \right|$$

(24) $$G_{APC}=\sum_{l=1}^{L}\sum_{k=1}^{m}X_{k,l}V_{k,l}((\sum_{e=1}^{n}x_{l,e}v_{l,e})/\left|Y_{l}\right|)-(1-f_{2})\sum_{l=1}^{L}\sum_{k=1}^{m}X_{k,l}V_{k,l}$$

where $X_{k,l}$ is the macrostructural design variable of the *k*th macroscale element at the *l*th layer and $x_{l,e}$ is the microstructural design variable of the *e*th element at the *l*th layer. $f_{1}$ is the specified structural coverage ratio, $f_{2}$ is the target average porosity ratio of all of the microstructures, and *L* is the total number of layers. $\left|\Omega \right|$ is the volume of the macroscopic design domain, and $\left|Y_{l}\right|$ is the volume of the element in the *l*th layer. *M* and *n* denote the total number of macroscale elements located in each layer and the microscale elements of each element, respectively. The symbol $V_{k,l}$ represents the volume of the *k*th macroscale element at the *l*th layer, and $v_{l,e}$ is the *e*th microscale element volume of the element in the *l*th layer. The actual material usage is $f_{1}(1-f_{2})\left|\Omega \right|$.

**Figure 9:**
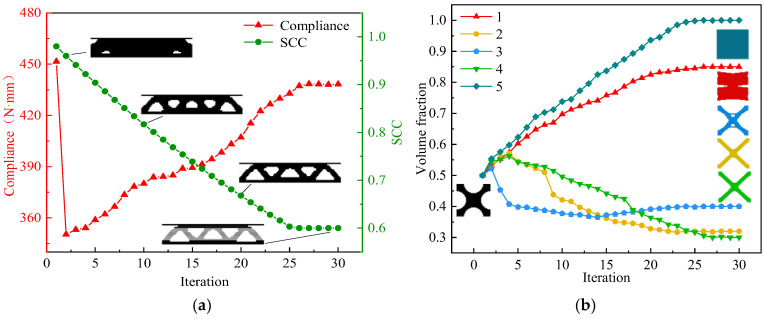
MMLG structure topology optimization history. (**a**) Macrostructure optimization history chart. (**b**) Microstructure optimization history chart.

The macroscopic sandwich structure is divided into five layers in this paper, which will produce five microstructures. The parameters set during the optimization process are as follows: SCC=0.6, GAPC=0.4, *P* = 3, *r_min_* = 3, and *er* = 0.02. Figure 9a,b show the optimization processes of the macrostructure and the microstructure.

**Figure 10:**
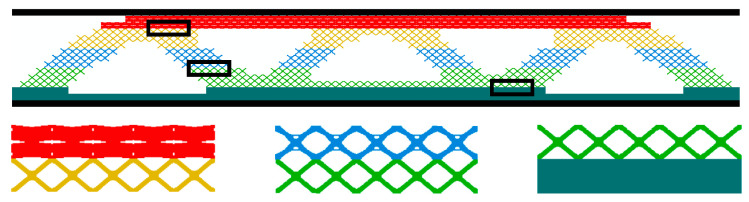
MMLG structure topology optimization result.

From Figure 9a, it shows that there is a significant decrease in macroscopic compliance from step 1 to step 2, which is similar to the reasons for this phenomenon in Section 4.1 and Section 4.2. In the subsequent iterations, the variation trends of volume fractions of several microstructures are different. As the SCC decreases, the macroscopic compliance gradually increases to 26 steps and then tends to stabilize. The iterations converge after 30 steps, and the compliance value ultimately reaches 438.52 N⋅mm. Figure 10 and Table 4 show the optimization results. Compared with the traditional sandwich structure, the core material of the layered sandwich structure is more uneven, so it is more difficult to manufacture. Many scholars have demonstrated through numerous numerical simulations [53] that the structure has greater stiffness and load carrying capacity when the density of the core layer on the loaded side or the restrained side is relatively higher [54]. These findings are fully consistent with the optimization results obtained in this paper.

### 4.4. Computational Efficiency Analysis of Optimization Algorithms

**Figure 11:**
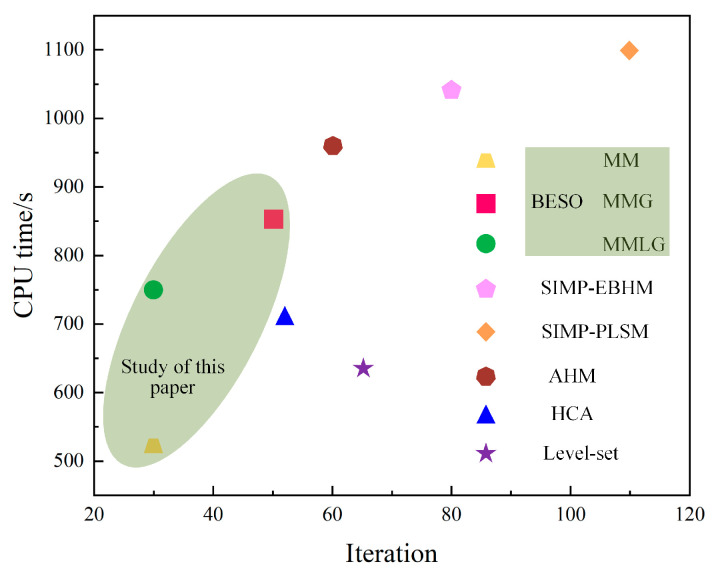
Comparison of efficiency of different multiscale concurrent topology optimization algorithms [31,38,50,51,52].

In order to consider the computational efficiency of the algorithm proposed in this study, it was compared with similar multiscale concurrent topology optimization algorithms in existing literatures. The optimization models all use the sandwich simply supported beam structure in this paper, with the same material properties and optimization objectives. The configuration of the computer used for optimization is as follows. The CPU is Intel Core i7-10700 (8 cores/16 threads) (Intel America). The CPU Clock Speed is 2.9 GHz. The RAM is 32 GB. The GPU is GTX 1050Ti (NVIDIA America). Comparing the number of iteration steps required by different optimization algorithms with the CPU running time is shown in Figure 11. It can be seen that compared with other algorithms, the multiscale concurrent topology optimization algorithm based on the BESO method proposed in this paper can effectively reduce the iterations and the total CPU computation time. This is a simple and efficient algorithm, which is more conducive to practical engineering applications.

### 4.5. Three Multiscale Concurrent Topology Optimization of Sandwich Cantilever Beam Under Uniform Distributed Load

**Figure 12:**
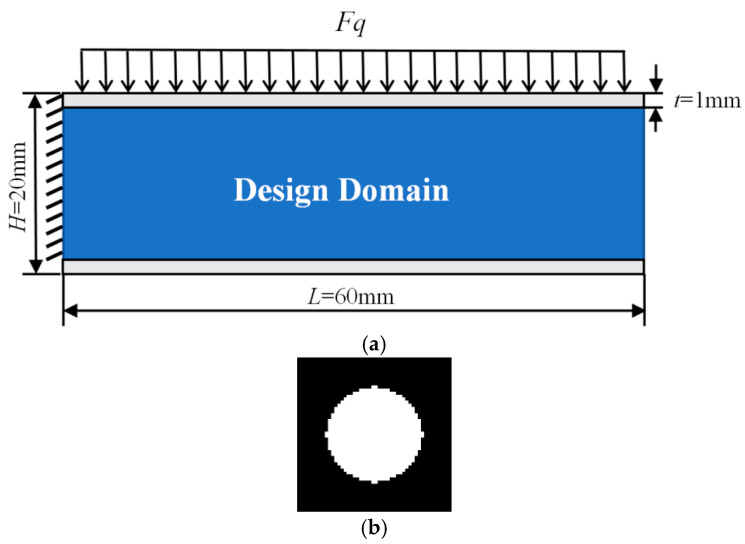
Optimization design and initial microstructure. (**a**) Initial optimization model of sandwich simply supported beam. (**b**) Initial microstructure.

A sandwich cantilever beam with length *L* = 60 mm and height *H* = 20 mm is shown in Figure 12a. The top and bottom panels are non-design domains, and the panel thickness *t* = 1 mm. The design domain is discretized into quadrilateral elements with a side length of 1 mm. The top panel is subjected to a uniform distributed load, *F_q_* = 30 N. The left edge of the cantilever beam is fixed. The initial microstructure is a rectangle with circular holes, which is shown in Figure 12b. The microstructure is discretized into 50×50 quadrilateral elements. The objective function is to minimize the macroscopic compliance. For comparison, three multiscale concurrent topology optimization structures have the same parameters: *Macrovolfrac* = 0.6, *Microvolfrac* =0.6, penalty index *P* = 3, filtering radius *r_min_* = 3, evolution rate *er* = 0.02.

**Figure 13:**
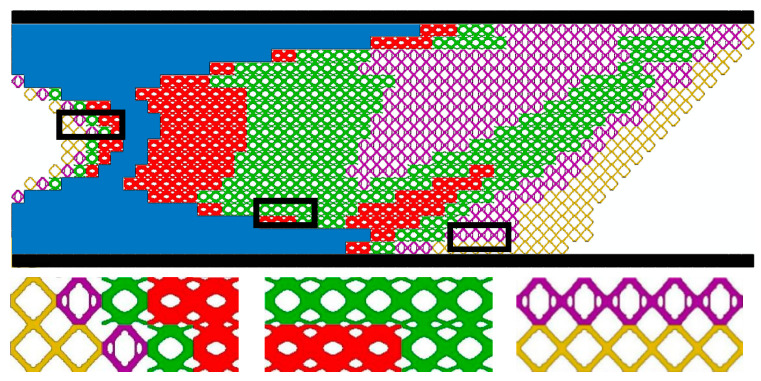
MMG structure topology optimization result.

**Figure 14:**
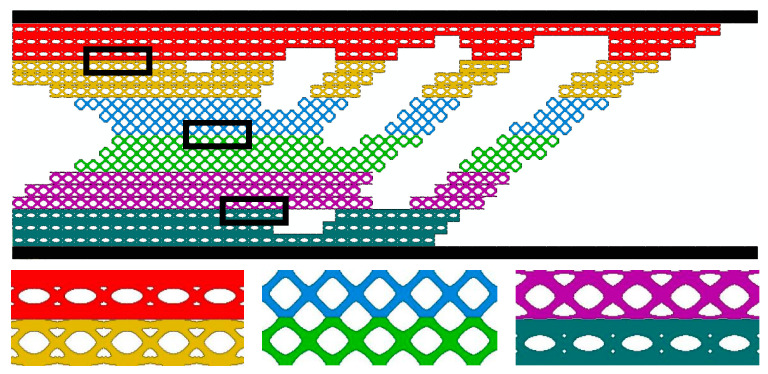
MMLG structure topology optimization result.

Figure 13 and Figure 14 and Table 5, Table 6 and Table 7 show the optimization results of the MM structure, the MMG structure, and the MMLG structure, respectively. The compliance is MM (C=412.57 N⋅mm), MMG (C=256.83 N⋅mm), and MMLG (C=352.91 N⋅mm). The stiffness of the three multiscale optimization results, in descending order, is the MMG structure, the MMLG structure, and the MM structure. It can be concluded that the gradient multiscale structure is superior to the uniform multiscale structure.

### 4.6. Three Multiscale Concurrent Topology Optimization Methods of 3D Sandwich Fully Clamped Beam Under Regional Uniform Distributed Load

**Figure 15:**
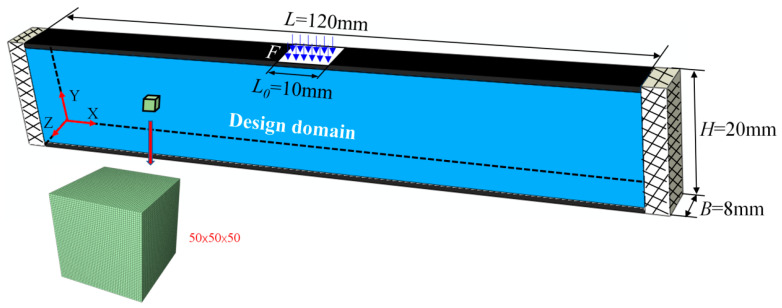
A 3D fully clamped beam structure.

**Figure 16:**
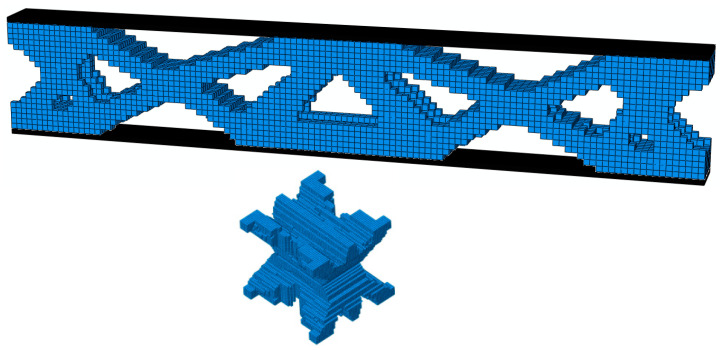
MM structure topology optimization result.

**Figure 17:**
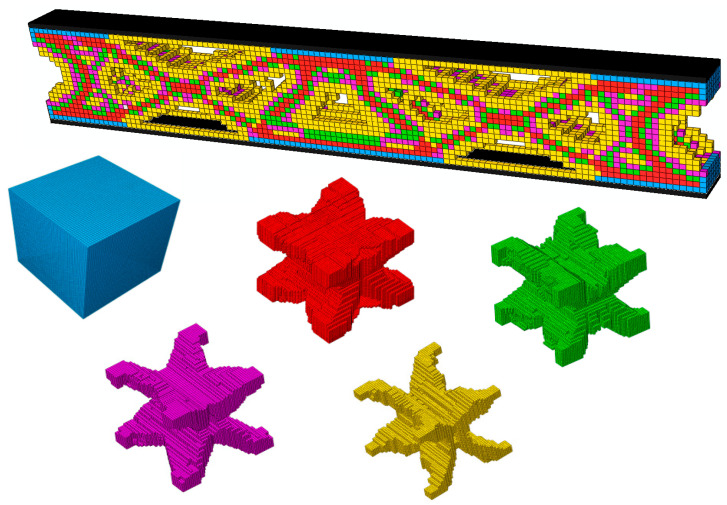
MMG structure topology optimization result (the volume fraction of five microstructures varies from large to small, with values of 1, 0.8, 0.6, 0.4, and 0.2).

**Figure 18:**
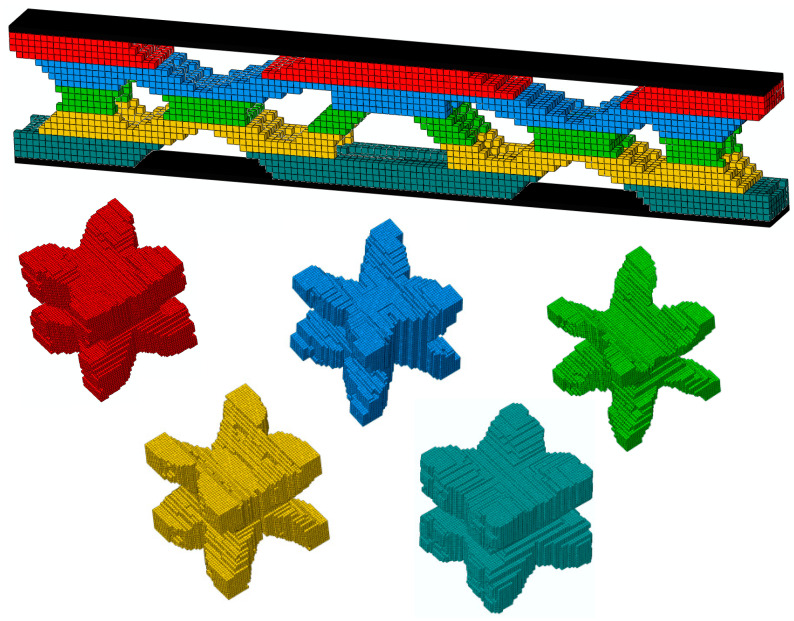
MMLG structure topology optimization result.

Similarly to the 2D case, three multiscale concurrent topology optimization methods can be easily extended to 3D structures [49]. Figure 15 shows a 3D sandwich fully clamped beam under a regional, uniform, distributed load. The detailed geometric parameters are length *L* = 120 mm, core layer height *H* = 20 mm, and width *B* = 8 mm, and the panel thickness *t* = 1 mm. The load application area is the central width of the top panel, *L*_0_ = 10 mm, *F* = 50 N. The microstructure is discretized into 50×50×50 hexahedron elements. The objection function is to minimize the macroscopic compliance. For comparison, three multiscale concurrent topology optimization structures have the same parameters: *Macrovolfrac* = 0.5, *Microvolfrac* = 0.6, penalty index *P* = 3, filtering radius *r_min_* = 3, evolution rate *er* = 0.02. The compliance is M (C=2593.41 N⋅mm), MMG (C=2081.72 N⋅mm), and MMLG (C=1884.36 N⋅mm). Figure 16, Figure 17 and Figure 18 show the results of optimization. This example illustrates the capability of the three multiscale optimization methods in 3D structures. At present, we have not conducted much research on 3D multiscale topology optimization, which is also the focus of our next work and future research.

## 5. Analysis of Experimental and Simulation Results

### 5.1. Preparing Test Samples

**Figure 19:**
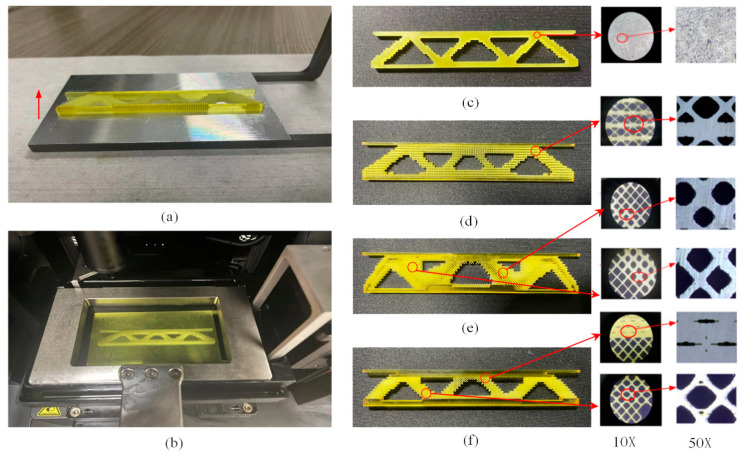
3D printing model preparation and results. (**a**) Printing direction. (**b**) Finished product status. (**c**) M structure. (**d**) MM structure. (**e**) MMG structure. (**f**) MMLG structure.

All structural specimens are prepared using the BMF Micro Fabrication NanoArch S140 3D high-precision stereo lithography appearance (SLA) printer (BMF Precision Tech Inc. Chongqing, China) for printing. Light yellow HD photosensitive resin material is used for printing, and the printing direction is the thickness direction (Figure 19a). To improve the printing accuracy, the single-layer printing thickness is controlled to be 20 μm, with a total of 200 layers printed. The light intensity is 80 lx, the single-layer exposure time is 5 s, and the single-layer liquid leveling time is 800 s. The dimensions of all specimens are 15 mm×9 mm×4 mm. Figure 19b shows the status of the printed product. As shown in Figure 19c–f, the finished print is placed under a microscope at 10× and 50× magnification to check the print quality.

**Figure 20:**
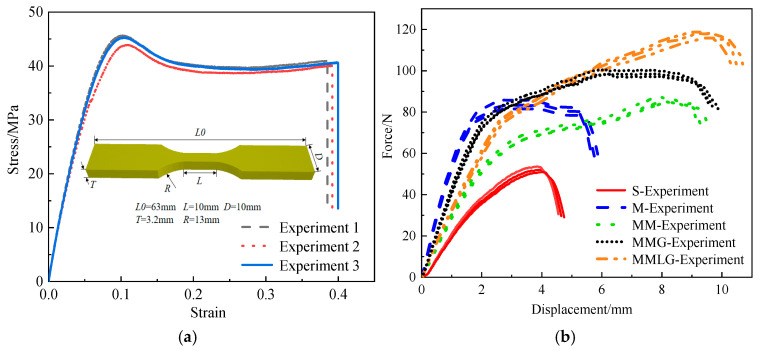
(**a**) Material stress–strain curve. (**b**) Load displacement curve of 5 structures.

To determine the mechanical properties of the HD photosensitive resin materials, dog-bone-shaped solid tensile test specimens in accordance with ASTM D638-10 standards for tensile testing of plastics were also 3D printed and subjected to quasi-static tension at a strain rate of $10^{-4}/s$. The true stress–strain curve is shown in Figure 20. Based on the data provided by the supplier, it can be concluded that density $\rho =1.2\;g/cm^{3}$, elastic modulus E=1.12 GPa, Poisson’s ratio μ=0.3, and the yield strength is 45.63 MPa.

### 5.2. Static Three-Point Bending Experiment

Five types of specimens are subjected to static three-point bending tests using the TCS-2000-GDL (Gotech Testing Machines Inc. Taiwan, China) hydraulic universal testing machine. The fixed loading rate is 0.1 mm/min, and the supported span is 8 mm. The load and displacement of the specimens are measured by the loading unit of the testing machine. To observe the deformation process of each structural specimen in the experiment, high-definition cameras are used to record the experimental process. As shown in Figure 20a, three repeated experiments are conducted on each structural specimen. The three sets of experimental data for each specimen are close to each other, so the experimental results are reliable and valid.

The finite element software ABAQUS is used for numerical simulation, and the computational model of five specimens is established. The model uses a four-node bilinear plane stress quadrilateral and reduced integration with an hourglass control element (CPS4R), and the material is selected from the data measured in the experiments in Section 4.1. The indenter and the support are set as rigid bodies, and the support is defined as a fully constrained boundary condition. The specimen is placed between the indenter and the support and adopts hard contact; the friction is 0.2. The indenter moves downward at a constant speed of 1 m/s [55]. (During the entire analysis process, the kinetic energy is indeed negligibly small compared to the internal energy, and the load displacement response is independent of the loading speed. In this case, simulation based on deformation at 1 m/s can be considered approximately quasi-static). The model grid size adopts an optimized structure with a minimum edge length size of 0.02 mm. In the finite element simulation, only the response of the structure within the elastic–plastic deformation is investigated, without considering the fracture or the failure of the structure.

**Figure 21:**
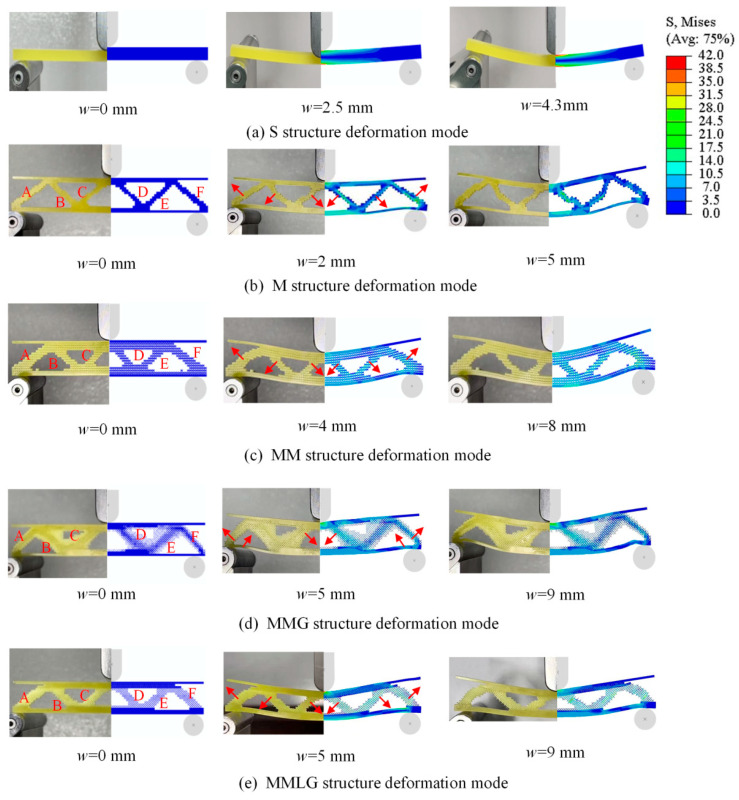
Comparison of experimental and numerical deformation modes of 5 structures.

Figure 20b shows the comparison of experimental and numerical simulation deformation modes for five different structures under different loading displacements. It can be seen that the results of the experiments and the simulations are in better agreement. Figure 21a shows the deformation of the S structure specimen under different displacements. When the loading displacement of the S structure increases from 0 to w=2.5 mm and w=4.3 mm, the bending degree in the middle of the specimen increases continuously. From the figure, the equivalent stress of the beam is higher at the upper and lower sides, and it gradually decreases towards the neutral layer. The distribution of elements with greater stress is similar to the distribution of solid materials in the initial stage of the MMG structural optimization process, which also indicates that the optimization is a process of removing inefficient elements and retaining efficient ones.

To simplify the description, the specimens in Figure 21b–e are divided into top and bottom panels and the middle core layer of the A, B, C, D, E, and F parts. Figure 21b shows the deformation of the M structure specimens under different displacements. As the loading displacement increases from 0 to w=2 mm, the structure reaches its elastic limit and begins to buckle. The middle of the top panel is deformed by downward compression bending, while the middle core layer is deformed in the direction of the arrow in the figure. The combined deformation of compression and bending occurs in the C, D regions, and the combined deformation of tension and bending occurs mainly in the B, E regions. The A, F regions are bent by the stretching effect to the outside of the oblique top. The above deformation state is more obvious when the displacement is w=5 mm.

Figure 21c shows the deformation of the MM structure specimens under different displacements. As the displacement increases from 0 to w=4 mm, both the top and bottom panels are bent and deformed downward. The macroscopic deformation pattern of the middle core layer part is similar to the M structure, with bending in the direction indicated by the arrow. When the displacement w=8 mm, the deformation degree of the core layer is more obvious. With the deformation of the macrostructure, obvious extrusion deformation occurs between microscale elements.

Figure 21d shows the deformation of the MMG structure specimens under different displacements. As the displacement increases from 0 to w=5 mm, both the top and bottom panels are deformed by downward compression bending. The area of the bottom panel between A, B and E, F will produce upward bending deformation after being squeezed due to its thinness. The middle core layers are less deformed and slightly bent in the direction indicated by the arrow. When the displacement w=9 mm, both the macrostructure and the microstructure are more deformed, while the smaller microscale elements with volume fractions of 0.2, 0.4 are more obvious.

Figure 21e shows the deformation of the MMLG structure specimens under different displacements. The deformation pattern is similar to that of the MM structure. When the displacement w=9 mm, the deformation of each part of the macrostructure is obvious. For the microscale, the extrusion deformation between the third and fourth “X”-shaped microstructures in the core layer are more intense.

**Figure 22:**
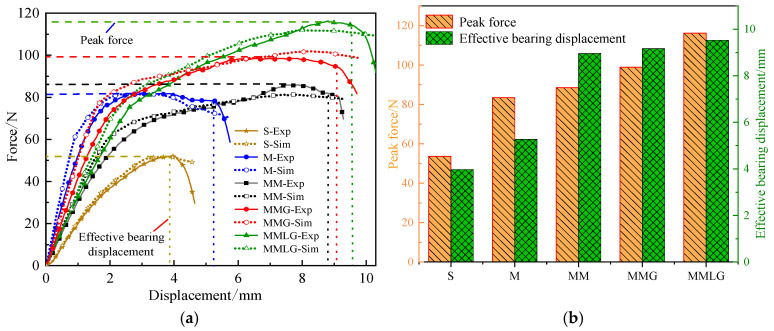
(**a**) Comparison of force–displacement curves between experimental and numerical values of five structures. (**b**) Comparison of peak force and effective bearing displacement of five structures.

As shown in Figure 22a, take the average of the three sets of force–displacement curves obtained from each specimen experiment and compare them with the simulation results. From the force–displacement curves of the simulation and the experiment, it can be seen that the curve variation trend matches well, which proves the effectiveness of the modeling and simulation methods used. It shows that the traditional single-scale macro optimized structure M has a higher stiffness value due to the absence of a microstructure, while the solid structure S has the smallest stiffness value. The stiffness of the MMLG structure is greater than that of the MM structure but lesser than that of the MMG structure. Due to the interaction of microstructures during the loading process, the core layer of the three types of multiscale structures enters the plastic strengthening stage after reaching the elastic limit, and the force–displacement curve shows an upward trend. The whole structure still has good load-bearing capacity. The M structure exhibits relatively stable platform section loads after reaching the elastic limit, while the S structure loses its stiffness and load-bearing capacity after buckling (specific deformation mechanism analysis is provided in Section 5.3).

The peak force and effective bearing displacement of the five structures are shown in Figure 22b. From high to low, the MMLG structure is 116.24 N, 9.53 mm; the MMG structure is 98.91 N, 9.17 mm; the MM structure is 88.57 N, 8.96 mm; the M structure is 83.47 N, 5.27 mm; and the S structure is 53.63 N, 3.98 mm. From the comparison, the peak force and the effective bearing displacement of the three multiscale optimized structures are greater than those of the other two comparison structures, which exhibit better bearing performance.

### 5.3. Macro and Micro Deformation Mechanisms and Energy Absorption of Structures

**Figure 23:**
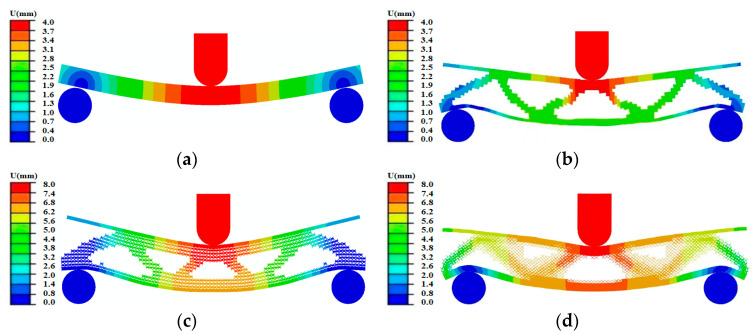

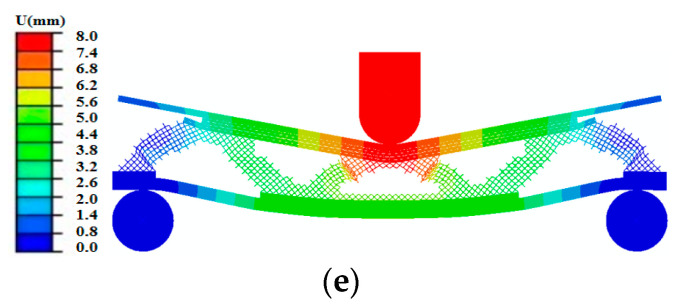
Displacement of 5 structures. (**a**) S structure specimen displacement. (**b**) M structure specimen displacement. (**c**) MM structure specimen displacement. (**d**) MMG structure specimen displacement. (**e**) MMLG structure specimen displacement.

Due to the different degrees of structural deformation, take the simulation results with large deformation during the loading processes of five structures for deformation analysis. The S structure w=4 mm, the M structure w=4 mm, the MM structure w=8 mm, the MMG structure w=8 mm, and the MMLG structure w=8 mm. Figure 23 shows the displacement profiles of the five structures and analyzes the macroscopic deformation of the five structures. From Figure 23a, the maximum deformation region of the S structure is located in the middle of the specimen directly below the indenter. From Figure 23b–e, the maximum deformation regions of the four optimized structures are the middle area of the top panel and the two support inclined arms in the middle of the core layer. The macroscopic whole deformation gradually decreases from the loading position in the middle of the specimen to the support ends on both sides.

**Figure 24:**
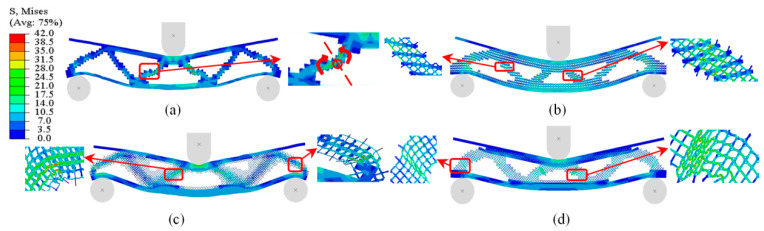
Deformation modes of 4 structural specimens: (**a**) M structure *w* = 4 mm deformation mode; (**b**) MM structure *w* = 7 mm deformation mode; (**c**) MMG structure *w* = 7 mm deformation mode; (**d**) MMLG structure *w* = 7 mm deformation mode.

The von Mises stress profiles of the four optimized structures are locally enlarged, and their deformation modes are analyzed. In Figure 24a, the support inclined arm of the middle core layer of the M structure produces local plastic deformation during the bending process. With the change in loading displacement, the upper and lower parts of the inclined arm rotate approximately around the plastic deformation region, while the other undeformed or slightly deformed parts make a rigid body motion at the macroscopic level. Thus, the macrostructure enters a relatively stable plateau phase after the elastic limit (Figure 22a). For the three types of multiscale optimized structural specimens, as shown in Figure 24b–d, the supporting inclined arms responsible for the main force transmission path of the core layer not only show bending deformation on the macrostructure but also exhibit obvious mutual contact and compression on the microstructure. The deformation of the elements with smaller volume fractions and finer rod diameters is more intense. This also leads to a better load-bearing capacity for the three multiscale optimized structures that enter the strengthening phase after the elastic limit.

For the sandwich structures, the specific energy absorption (SEA) and the energy ratio of the core layer are an important evaluation index for the energy absorption characteristics of the structure. SEA indicates the energy absorbed per unit mass of the structure, and the higher its value, the better the energy absorption capacity of the structure. The formula for SEA is

(25) $$SEA=\frac{E_{A}}{M}$$

where *M* is the mass of the structure and $E_{A}$ is the energy absorbed during the bending process of the structure, which can be expressed as

(26) $$E_{A}=\int_{0}^{w}F(x)dx$$

where w is the displacement of the structure and F(x) is a function of the force displacement curve. The energy ratio of the core layer is the ratio of the plastic energy dissipation of the core layer to the plastic energy dissipation of the overall structure ($E_{c}/E_{w}$). The larger the energy ratio of the core layer, the more excellent the material distribution of the optimized structure, and the better the energy absorption effect of the sandwich structure.

**Figure 25:**
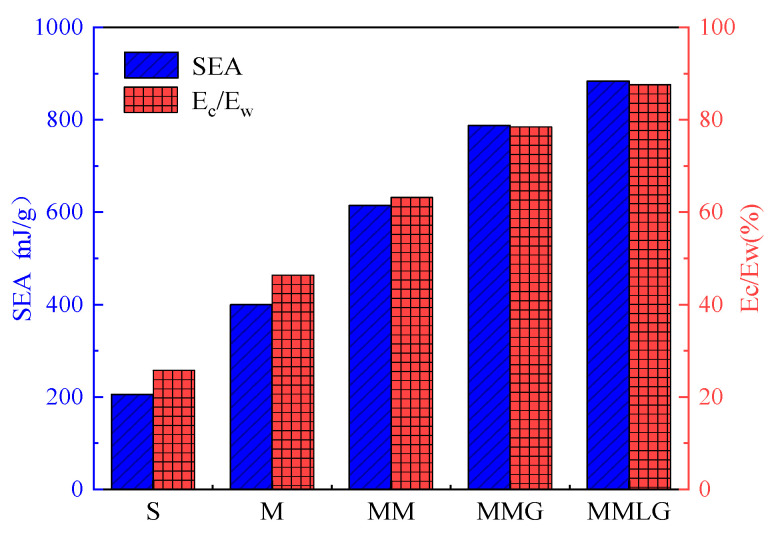
Specific energy absorption and energy absorption proportion of 5 structures.

From the structural deformation diagram in Figure 21, it can be seen that under the same volume constraint and load conditions, the energy absorbed by the core layer is significantly greater than that of the top and bottom panels due to the compression of the core layer of the four optimized structures. Due to the presence of the microstructure, the three types of multiscale optimization structures in the core layer region are more prone to bending or compression deformation between microscale elements under load. So, the compression areas of the core layers are relatively larger, resulting in greater plastic deformation of the overall structures and better energy absorption capacity. Figure 25 shows the simulated energy absorption and core layer energy ratio of five different structures. The SEA of the five structures, from high to low, is as follows: the MMLG structure, the MMG structure, the MM structure, the M structure, and the S structure. And, the MMLG structure is 10.77%, 30.45%, 54.77%, and 76.7% higher than the other structures, respectively. The proportion of core layer energy from high to low is the MMLG structure at 87.6%, the MMG structure at 78.5%, the MM structure at 63.2%, the M structure at 36.4%, and the S structure at 25.8%.

## 6. Conclusions

In this paper, with the maximum structural stiffness as the optimization objective, a multiscale topology optimization method with the framework of BESO is established. The information interaction between MATLAB and ABAQUS is also used to simplify the optimization process to improve the efficiency of operations. From the optimization results, the overall optimization process is stable, and the optimization structure is clear and reasonable. The three multiscale optimized structures (the MM structure, the MMG structure, and the MMLG structure), as well as the M structure and the S structure as the comparison groups, were printed using a high-precision 3D printer. Static three-point bending experiments and finite element numerical simulations were also performed on all specimens.

Based on the experimental and numerical simulation results, the deformation mechanism and the mechanical properties of the five structures were analyzed, and the following conclusions were obtained. Under the same volume fraction and loading conditions, the stiffness value of the MMG structure is only smaller than that of the M structure and larger than that of the other structures. The MMLG structure has superior ultimate load-bearing capacity and energy absorption characteristics. The MM structure composed of a single microstructure has a lower load-bearing capacity and energy absorption capacity than the two gradient structures, and it is better than the comparison structure. This also demonstrates that compared with the traditional macrostructure topology optimization methods, the multiscale topology optimization method in this paper provides a different new design idea so that materials can be allocated in different ways. By increasing the consideration of the microstructure, multiscale concurrent topology optimization opens up a wider design space. The structure can be further optimized and improved.

Finally, through multiscale concurrent topology optimization of the sandwich cantilever beam under a uniform distributed load and the 3D sandwich fully clamped beam under a regional, uniform, distributed load, the correctness and general applicability of the proposed optimization method are demonstrated.

However, this method has its shortcomings as well, such as high computational costs and the existence of an optimized structure with uneven, jagged edges. We will combine the actual application of engineering and further research on the existing problems.

## Figures and Tables

**Table 1:** Classification of displacement vectors.

Displacement Number	Displacement
$U_{1}$	Displacement of the four vertices A, B, C, D
$U_{2}$	Internal node displacement of the cell
$U_{3}$	Divide the displacement of the vertex of the edge 1, 4
$U_{4}$	Divide the displacement of the vertex of the edge 2, 3

**Table 2:** MM structure topology optimization result.

MM structure concurrent topology optimization result	Equivalent elastic modulus $D^{H}$
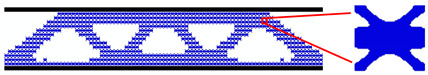	$\left(\begin{array}{ccc} 1.6676 & 0.3531 & 0\\ 0.3531 & 0.4974 & 0\\ 0 & 0 & 0.3982 \end{array}\right)$

**Table 3:** Microstructures of MMG structure.

Volume Fraction	0.2	0.4	0.6	0.8
Microstructure	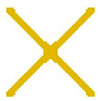	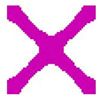	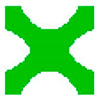	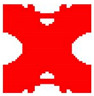
Equivalent elastic modulus $D^{H}$	$\left(\begin{array}{ccc} 0.2372 & 0.2311 & 0\\ 0.2311 & 0.2481 & 0\\ 0 & 0 & 0.2127 \end{array}\right)$	$\left(\begin{array}{ccc} 0.9519 & 0.3563 & 0\\ 0.3563 & 0.4442 & 0\\ 0 & 0 & 0.3493 \end{array}\right)$	$\left(\begin{array}{ccc} 1.6296 & 0.6415 & 0\\ 0.6415 & 1.0031 & 0\\ 0 & 0 & 0.6356 \end{array}\right)$	$\left(\begin{array}{ccc} 3.1726 & 0.9554 & 0\\ 0.9554 & 2.5322 & 0\\ 0 & 0 & 1.0616 \end{array}\right)$

**Table 4:** Microstructures of MMLG structure.

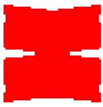	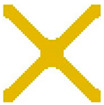	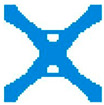
$\left(\begin{array}{ccc} 3.7414 & 0.5769 & 0\\ 0.5769 & 1.6345 & 0\\ 0 & 0 & 0.8109 \end{array}\right)$	$\left(\begin{array}{ccc} 0.4294 & 0.3911 & 0\\ 0.3911 & 0.4224 & 0\\ 0 & 0 & 0.3495 \end{array}\right)$	$\left(\begin{array}{ccc} 0.5877 & 0.4548 & 0\\ 0.4548 & 0.4655 & 0\\ 0 & 0 & 0.3939 \end{array}\right)$
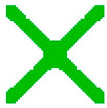	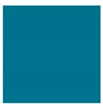	
$\left(\begin{array}{ccc} 0.4161 & 0.3815 & 0\\ 0.3815 & 0.4095 & 0\\ 0 & 0 & 0.3399 \end{array}\right)$	$\left(\begin{array}{ccc} 4.4660 & 1.1754 & 0\\ 1.1754 & 3.8386 & 0\\ 0 & 0 & 1.4562 \end{array}\right)$	

**Table 5:** MM structure topology optimization result.

MM structure concurrent topology optimization result	Equivalent elastic modulus $D^{H}$
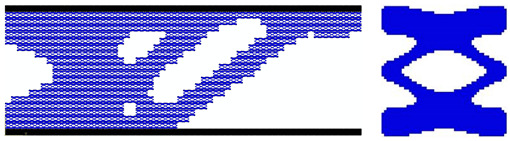	$\left(\begin{array}{ccc} 1.7185 & 0.6742 & 0\\ 0.6742 & 1.1248 & 0\\ 0 & 0 & 0.7133 \end{array}\right)$

**Table 6:** Microstructures of MMG structure.

Volume Fraction	0.2	0.4	0.6	0.8
Microstructure	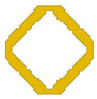	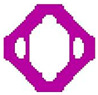	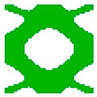	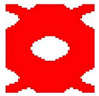
Equivalent elastic modulus $D^{H}$	$\left(\begin{array}{ccc} 0.2372 & 0.2311 & 0\\ 0.2311 & 0.2481 & 0\\ 0 & 0 & 0.2127 \end{array}\right)$	$\left(\begin{array}{ccc} 0.9519 & 0.3563 & 0\\ 0.3563 & 0.4442 & 0\\ 0 & 0 & 0.3493 \end{array}\right)$	$\left(\begin{array}{ccc} 1.6296 & 0.6415 & 0\\ 0.6415 & 1.0031 & 0\\ 0 & 0 & 0.6356 \end{array}\right)$	$\left(\begin{array}{ccc} 3.1726 & 0.9554 & 0\\ 0.9554 & 2.5322 & 0\\ 0 & 0 & 1.0616 \end{array}\right)$

**Table 7:** Microstructures of MMLG structure.

Microstructure	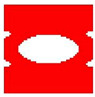	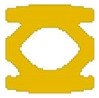	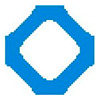
Equivalent elastic modulus $D^{H}$	$\left(\begin{array}{ccc} 3.1425 & 0.6529 & 0\\ 0.6529 & 2.4315 & 0\\ 0 & 0 & 0.9037 \end{array}\right)$	$\left(\begin{array}{ccc} 2.6468 & 0.5237 & 0\\ 0.5237 & 1.5329 & 0\\ 0 & 0 & 0.7246 \end{array}\right)$	$\left(\begin{array}{ccc} 1.1376 & 0.3725 & 0\\ 0.3725 & 0.5312 & 0\\ 0 & 0 & 0.4109 \end{array}\right)$
Microstructure	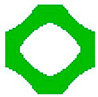	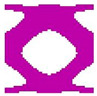	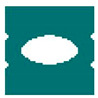
Equivalent elastic modulus $D^{H}$	$\left(\begin{array}{ccc} 1.1436 & 0.4019 & 0\\ 0.4019 & 0.5445 & 0\\ 0 & 0 & 0.4217 \end{array}\right)$	$\left(\begin{array}{ccc} 2.5974 & 0.5126 & 0\\ 0.5126 & 1.4802 & 0\\ 0 & 0 & 0.7741 \end{array}\right)$	$\left(\begin{array}{ccc} 3.4574 & 0.7238 & 0\\ 0.7238 & 2.6742 & 0\\ 0 & 0 & 0.9146 \end{array}\right)$

## Data Availability

The data presented in this study are available on request from the corresponding author due to some of the data involves privacy.
